# The Effect of a Community-Based Complementary Feeding Education Program on the Nutritional Status of Infants in Polokwane Municipality, Limpopo Province, South Africa

**DOI:** 10.3390/children11121425

**Published:** 2024-11-26

**Authors:** Maishahataba Solomon Makwela, Lindelani Fhumudzani Mushaphi, Lufuno Makhado

**Affiliations:** 1 Department of Human Nutrition and Dietetics, Faculty of Health Sciences, University of Limpopo, Polokwane 0727, Limpopo Province, South Africa; 2 Department of Nutrition, Faculty of Health Sciences, University of Venda, 1 University Road, Thohoyandou 0950, Limpopo Province, South Africa; lindelani.mushaphi@univen.ac.za; 3 Department of Public Health, Faculty of Health Sciences, University of Venda, 1 University Road, Thohoyandou 0950, Limpopo Province, South Africa; lufuno.makhado@univen.ac.za

**Keywords:** anthropometry, nutritional status, complementary feeding

## Abstract

Background: Appropriate complimentary feeding (CF) has the potential to improve growth and development in infants from 6 months to 2 years of age. This study’s aim was to assess the effect of a CF education program on the nutritional status of infants aged 3–12 months in the Polokwane Municipality. Methods: A longitudinal (non-randomized), quasi-experimental intervention study was conducted among 187 caregivers. The caregiver-infant pairs (CIPs) in the intervention group (*n* = 95) received CF education and a six-month telephone follow-up support. The CIPs in the control group (*n* = 94) received no intervention. We collected data at baseline and end line using structured questionnaires. Intervention effectiveness was evaluated by comparing mean changes in anthropometric measurements between intervention and control groups using difference-in-difference analysis. Per protocol, analysis was run. Results: The results show that the children in the intervention group gained more weight after the intervention, were taller, and the mid-upper arm circumference (MUAC) increased more than in the control group. At baseline, the weight of infants was 7.37 ± 0.83 and 6.69 ± 0.13 kg in the intervention and control. At end line, significant weight and length gain were observed in the intervention group (difference-in-difference (DID) = 1.82 kg, *p* < 0.001) and (DID = 7.78 cm, *p* < 0.01), respectively. The intervention group showed significant gain in MUAC of 1.68 cm (*p* = 0.047), with no noticeable effect on the head circumference at end line; DID (0.16 cm; *p* = 0.950). Conclusions: Community-based nutrition intervention programs can effectively improve the anthropometric nutritional status of children aged 3–12 months.

## 1. Introduction

Exclusive breastfeeding (EBF) is defined as the feeding of an infant with only breast milk and no additional food, water, or other liquids (except for medicines and vitamins, if needed) during the first six months of life [1]. During this period (0–6 months), breast milk has the perfect balance of nutrition and antibodies for growth and immune support [2]. However, the nutritional potency of breast milk decreases post the 6-month mark with a noted deficiency in vitamin A, B6, iron, and zinc, making it challenging to meet the growing infants’ needs [3]. Complementary feeding is required when the infant’s energy and nutritional needs exceed what EBF can supply [4]. According to the World Health Organization (WHO), complementary feeding should be introduced when the infant reaches six month of life, with the continuation of breastfeeding for a minimum of two years [5]. Complementary feeding (CF) entails a period wherein the infants’ diet changes from EBF/formula to the gradual introduction of solid/semi-solid food [5]. Complementary feeding, when correctly implemented, offers the opportunity for the prevention of stunting, wasting, being overweight, and obesity and to improve long-term health and growth [6]. Breastfeeding or replacement feeding (with formula or animal milk) should continue for as long as two years or longer, while infants should start receiving nutritionally appropriate and acceptable complementary foods at six months to meet their increasing nutritional demands [5].

Factors such as food insecurity negatively affect the implementation of complementary feeding and, subsequently, the baby’s nutritional status [7]. In South Africa, only 23% of children ages 6 to 23 months have met the criteria for a minimum acceptable diet [8]. Malnutrition among children under five is linked to inadequate complementary feeding practices, such as introducing semi-solid foods too early, poor hygiene, and providing foods lacking in essential nutrients [8]. These practices play a major role in an infant’s development of illnesses such as diarrhea, malnutrition, poor growth, infections, and impaired mental development [9]. In rural and low-income communities of South Africa, limited access to healthcare, inadequate dietary practices, and a lack of nutritional knowledge among caregivers exacerbate the risk of poor health outcomes, making it imperative to address these challenges through education and improved access to resources.

Education is recognized as an effective approach to improving CF practices, with studies showing that it can significantly enhance caregivers’ knowledge and practices, leading to better nutritional outcomes for children [9]. Complementary feeding education was associated with improvement in caregivers’ knowledge; if applied appropriately, it translates to practices that help provide adequate nutrition to the infant. A study in Indonesia found that intervention in the form of nutrition education had a significant effect on body weight for age [9]. However, a study in Vhembe district, Limpopo province, showed no effect of nutrition education programs on indicators of nutritional status [10]. In the same way, a study carried out in the province of Limpopo found no correlation between schooling programs and the anthropometric status of children under five [10].

Despite the critical role of proper feeding practices in ensuring the nutritional health of infants, improper feeding remains a significant problem in developing countries, including South Africa [11]. In the Limpopo Province, specifically in Polokwane Municipality, limited studies have explored the impact of nutrition education on infant feeding practices. Those that exist have primarily relied on lecture-based methods, neglecting the practical demonstration of preparing affordable, accessible, and culturally accepted foods using locally available ingredients. By evaluating the impact of a community-based supplemental feeding education program (which included telephonic follow-up and demonstrations) on the nutritional status of infants in Polokwane Municipality between the ages of 3 and 12 months, this study seeks to close these gaps and aid in the development of long-term plans to lower infant malnutrition in South Africa. The 6 months of telephone support involved knowledge refreshment, offering clarity where participants needed it and following up on the implementation and practice of the intervention knowledge. These telephonic follow-ups were conducted every two weeks.

## 2. Methodology

### 2.1. Research Design and Setting

**Figure 1:**
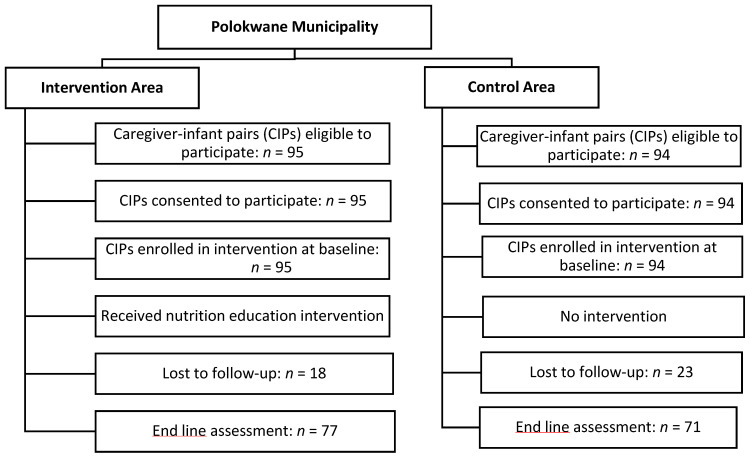
**Flow** chart showing the study design.

It was a quasi-experimental study design. Additionally, because data was gathered at two distinct reference points (baseline and end line), the study was longitudinal. The primary outcome of the present study was to evaluate the influence of a community-based complementary feeding education program on the nutritional status of infants aged 3 to 12 months. While the secondary outcome was to evaluate how effective the community based complementary feeding education interventions were on the nutritional status of the infant by measuring the change (baseline versus end line) in selected anthropometric measurements such as weight, length, mid upper arm circumference in the intervention group compared to the control group (Received no intervention). DIMAMO villages are located west of Polokwane, while Moletji is in the east. Simple random sampling by location was used to determine which area should serve as intervention or control. Moletji was assigned tail and DIMAMO as head. Tail and head represent the intervention and control groups, respectively. The coin was tossed, and caregiver-infant pairs in DIMAMO were enrolled as controls while Moletji served as the intervention group. A pretest–posttest control group design was used in the investigation. Prior to intervention, baseline data was gathered for both the control and intervention groups. After baseline, the control group did not receive complementary feeding education, while the intervention group did receive complementary feeding education. The intervention was carried out between September 2023 and February 2024. Following the intervention, data was gathered once more from both groups. Anthropometric nutritional status was reviewed following the six-month implementation of the intervention program. Polokwane municipality, in South Africa’s far north, is where this investigation was carried out. Approximately thirty percent of caregivers lived in rural villages governed by tribal authorities. The study design flow chart is shown in Figure 1, presented in the flow diagram is the recruitment process. These include the number of caregiver-infant pairs that were found to be eligible for the present study in both the intervention and control groups, the number of participants who consented to participate, those lost to follow-up, and those who managed to participate at both baseline and end line assessment.

### 2.2. Population and Sampling of the Study

**Figure 2:**
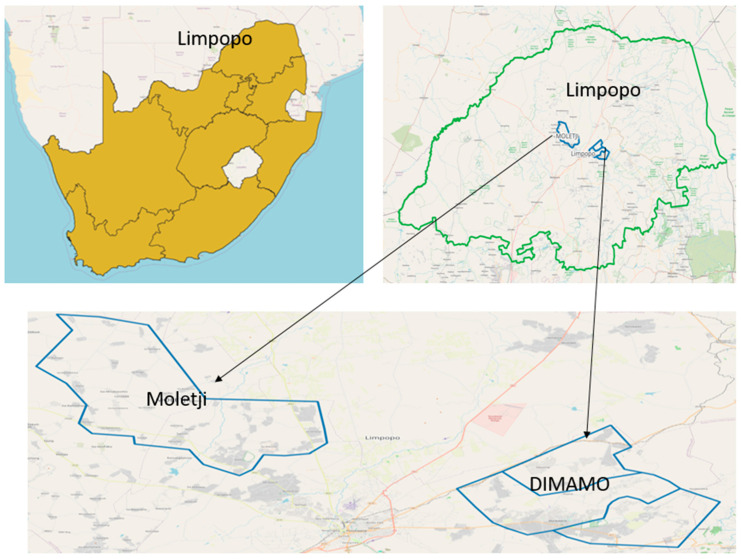
Study area. Created using Quantum Geographic Information System software (Q-GIS) [14].

The study population included caregivers and children aged 3 to 12 months residing in villages that fall under two tribal authorities, namely, Moletji and DIMAMO, which are in the Polokwane municipality of the Limpopo Province (Figure 2). According to clinic registers and the DIMAMO population demographics and surveillance system, there were approximately 158 and 146 children aged 3 to 12 months in the Moletji and DIMAMO tribal authorities, respectively. Therefore, 304 was regarded as the population size. For this study, ‘caregiver’ refers to the legal guardian of the child, who were mostly (97.9%) the biological mother of the child. The study was limited to caregivers who were at least 18 years old, spoke Sepedi and English, and were able to give informed consent. The sample size from an estimated population of 304 children was determined using Slovin’s formula [12] (Slovin, 1960 [13]): *n* = *N*/(1 − *Ne*^2^), where *n* is the sample size, *N* is the population size, and *e* is the acceptable margin of error (0.05). With 10% attrition added, the sample size was 171, for 189 caregivers.

### 2.3. Instruments and Data Collection

Eight (8) villages (4 experiment (E), 4 control (C)) from Polokwane Municipality in the Capricorn district of Limpopo, South Africa, were chosen by simple random sampling. All households with children who satisfied the inclusion criteria and agreed to participate were included in the study. About 189 (95 interventions and 94 control) children aged 3 to 12 months were included at baseline. Post-intervention, 77 children and 71 caregivers completed the study protocols and questionnaire. The drop in sample size was owing to a loss to follow-up, i.e., roughly 18 individual pairs in the intervention group and 23 in the control group. This equates to a 78% response rate for both children and caregivers. Complementary feeding knowledge and practices of caregivers and the nutritional status of infants were assessed.

Data were collected using a questionnaire that measured socio-demographic characteristics, anthropometric indices (weight, length, height, and mid–upper-arm circumference), complementary feeding knowledge, and practices. A questionnaire was administered during an oral interview with caregivers.

At baseline, the researcher and two trained field workers conducted interviews with participating caregivers on nutrition knowledge and feeding practices in the local language, Sepedi. Caregivers answered 49 knowledge questions and 20 practice questions. Using conventional techniques outlined by Lee and Nieman, the trained fieldworkers also obtained anthropometric measurements (weight, length, head circumference, and MUAC) that same day. The measurements were taken twice with a calibrated equipment ensuring children wore light clothes and were without shoes. The length was measured to the nearest 0.1 cm using the Seca measuring length board (model 417), and weight was measured to the nearest 0.01 kg on a portable battery-operated scale manufactured by Seca in Hamburg, Germany. A known weight was used to calibrate the scale prior to taking measurements. MUAC was carried out using color-coded, non-stretchable MUAC tape-child manufactured by Delta Surgical SA(PTY) LTD; the measurements were taken to the nearest 1 mm [12]. The complementary feeding education program (CFEP) was based on the messages from the South African Road-to-Health Booklet, the Infant and Young Child Feeding Policy (2013) [15], the Guiding Principles for Complementary Feeding of the Child (2023), and the Community Infant and Young Child Feeding Counselling Package (UNICEF), which was also used. The CFEP comprised 10 main topics relating to breastfeeding and complementary feeding (Table S1). These were discussed with the experimental group over 6 months. The experimental group was visited biweekly during the implementation phase for 25–30 min discussions.

#### 2.3.1. Implementation of the Intervention

During the intervention, 15–20 participants were gathered in a venue in the area. The researcher, who is a dietitian, led each session. Caregivers were led into each activity based on the training schedule (Table S3). Lectures were presented, followed by group discussions and back-and-forth demonstrations of recipes by facilitators and caregivers. A conceptual framework was developed to guide this study (Figure S1), which assumes that delivering complementary feeding education coupled with preparation/demonstration of recipes may improve caregivers’ infant feeding knowledge and practices, thereby improving the nutritional status of infants. During the intervention, preparation of complementary foods was conducted through demonstration of the 12 developed recipes. Recipes were developed using locally available, accessible, and culturally acceptable food (Table S4). The intervention fidelity checks were affected by conduction recap, encouraging participants to be actively involved during group discussions and observing child feeding after cooking sessions. After the intervention, telephone follow-ups were performed on individual participants for at least once a month. These telephone follow-ups took place for a period of six months. Telephone support involved reassurance, addressing challenges in food preparation, feeding, and offering clarity when needed.

#### 2.3.2. Change in Knowledge

Caregivers’ knowledge on infant feeding and practices were measured at baseline and followed up in both the intervention and control groups. The following questions were employed: What is exclusive breastfeeding? Age until exclusive breastfeeding ends? Benefits of exclusive breastfeeding? Age of introduction of complementary feeding? Actions to encourage infants to eat? How can you ensure good hygiene during the preparation of food? Why is good hygiene important in children? How to ensure safe preparation of infant food? Importance of washing hands with soap before feeding the infant? Conditions for washing hands? Appropriate storage of infant foods? What is the quantity of food to start feeding an infant six to eight months? What are the food consistencies suitable for children from six months? Food consistencies for children at eight months? Food consistencies for children at 12 months? Foods to avoid during the introduction of finger foods? Consequences of feeding food with incorrect consistency? At which month is the child expected to eat solid foods consumed at home? Consequences of delaying introducing lumpy food beyond 10 months? A score was generated at baseline and follow-up. The mean difference between the two was used to measure the change in knowledge in both groups.

### 2.4. Data Analysis

Data were analyzed by biostatistician researchers from the Research Development of the University of Limpopo (UL) and statisticians from the Department of Statistics and Computational Sciences at the University of Venda (UNIVEN) using the Statistical Package for Social Sciences version 27 (SPSS) and STATA 18. The Shapiro–Wilk Test was used to test for normality, and a *p*-value of more than 0.05 indicated that the data was normally distributed. A comparison of the caregiver’s characteristics of both the intervention and control groups was conducted at baseline. Relating to infants, anthropometric status in control and experiment groups were analyzed for both baseline and end line. Continuous data were expressed using mean and standard deviation. Categorical data were presented in terms of frequencies and percentages *n* (%). The difference-in-difference (DID) analysis was used to measure the effect of the intervention on knowledge and anthropometric indicators. The difference-in-difference test was found to be most relevant for the present study’s analysis for its ability to assess the causal impact of intervention and for its ability to be used where randomization is not fully possible [16]. For both descriptive and DID analysis, a *p*-value of less than 0.05 was considered significant. Per protocol analysis was run instead of the intention to treat.

In this study, knowledge was graded using Bloom’s cut-off for knowledge of 0–100% score and on a scale from poor to good. Poor knowledge is defined as receiving a total score between 0 and 50%, fair knowledge between 51 and 80%, and good knowledge between 81 and 100%. Anthropometric measurements were interpreted using WHO simplified tables for cut-off points for length-for-age z-scores (LAZ), weight-for-length z-scores (WLZ), and weight-for-age z-scores (WAZ) [17]. With reference to WHO (2008), the Z-score classification cut-off points were ≤−2 SD, which was used for W/A, W/L, and L/A. These imply that underweight, wasting, and stunting, respectively. Severe wasting, severe underweight, and severe stunting are classified according to the cut-off criterion of ≤−3 SD [17]. Furthermore, the cut-off points for weight for age of <+3 SD is considered overweight, while >3 SD is considered obesity.

### 2.5. Ethical Issues

The Human and Clinical Trials Research Ethics Committee (HCTREC) of The University of Venda, which issued the clearance certificate number FHS/22/NUT/12/2111, approved the study. Each study participant signed a formal informed consent form. Participants were told that they could withdraw from the study at any time without facing any repercussions. Participants’ confidential information was also kept private and confidential.

## 3. Results

The present study is aimed to assess the effect of complementary feeding education programs on the nutritional status of infants. Table 1 presents baseline characteristics of participants divided into the intervention and control groups. In the present study, there were 95 participants in the intervention group and 94 participants in the control group. No significant differences were observed between the groups in terms of sex (*p* = 0.157), marital status (*p* = 0.195), education level (*p* = 0.587), occupation (*p* = 0.978), type of housing (*p* = 0.654), and source of drinking water (*p* = 0.054). Most caregivers in both groups were females and unmarried (intervention = 70.5%; control = 78.7%); however, this was not significant. Almost a quarter (24%) of caregivers in the intervention group had achieved tertiary education compared to 18% in the control group. A significant difference was observed in the two groups, where almost 70% of participants in the control group reported that they often or sometimes did not have enough food to eat (*p* = 0.002). With regards to waste/rubbish disposal, three-quarters (75%) of caregivers had disposed of rubbish at the corner of their yards as compared to almost two-thirds (59%) in the control group; the difference was significant, *p* = 0.048. In both groups, the majority gave birth through normal delivery (intervention = 64%; control = 80%). However, it was noteworthy that more than a third (35%) of infants in the intervention area were delivered via Caesarean section, and the difference was statistically significant, *p* = 0.007.

**Figure 3:**
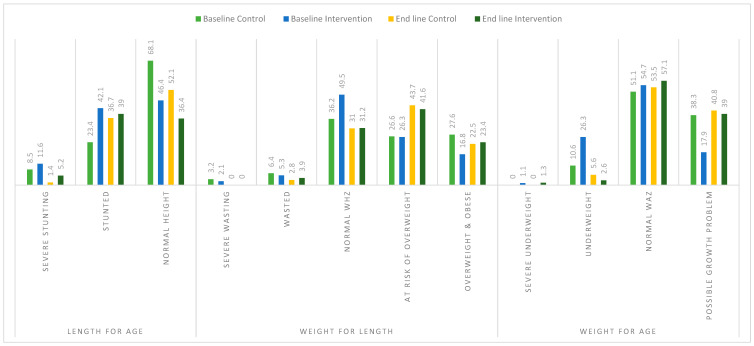
Anthropometric status of infants in the control and experiment groups at baseline and end line. Data is presented in terms of percentages analyzed using the cross tabs option in SPSS. WHZ: weight-for-height Z-score. WAZ: weight-for-age Z-score.

Figure 3: At baseline, there were no significant differences in length for age (*p* = 0.108) and weight for length (*p* = 0.451) of infants in the two groups. However, weight for age differed significantly between intervention and control groups (*p* = 0.017), where more than a quarter of infants in the experiment group were underweight as compared to the control group (26.3% versus 10.6%). Post-intervention: no significant changes in length-for-age Z-scores (LAZ), weight-for-age Z-scores (WAZ), and weight-for-length Z-scores (WLZ). However, severe stunting reduced from 11.6% to 5.2% in the intervention group (difference of 6.4%), stunting reduced from 42.1% to 39% (difference of 3.1%), while underweight reduced from 26.3% to 2.6% (difference of 23.7%) as compared to the control group. Detailed results are further presented in the Supplementary File (Table S2).

Infants’ mean weight, length, head circumference, and mean MUAC were measured at baseline and end line in both groups. The mean difference in the infants’ growth (weight, length, head circumference, and MUAC) between the intervention and control groups was used to calculate the effectiveness of the intervention. Weight and length grew by 1.85 kg and 4.85 cm, respectively, in the control group and 3.67 kg and 12.6 cm, respectively, in the intervention group. With a *p*-value of ≤0.001 for weight increase and *p* ≤ 0.001 for length, the differences between the control and intervention groups were significant. In both the intervention and control groups, there was no discernible difference in the change in head circumference between the baseline and end line (*p*-value = 0.950). The significant change (*p* = 0.047) for MUAC suggests that the intervention had an impact on mid-upper arm circumference.

The mean knowledge of caregivers was calculated in both groups at baseline and end line and the effectiveness of the intervention was measured by calculating the difference-in-difference in the mean change knowledge score between the intervention and control groups (Table 2). At baseline, the mean knowledge score for the control group was 29.70 ± 0.83, while that for the treatment group was 25.10 ± 0.97. At end line, the mean knowledge score for the control group was 25.0 ± 0.99, while for the treatment group it was 38.10 ± 0.31. The mean improvement in the control group’s knowledge score was −4.3, which means the average knowledge score for the control group decreased. For the experimental group, the improvement in the average score is 13.0 units. The difference in mean change in knowledge between the intervention and control groups was 17.72, which was found to be statistically significant (*p* = 0.001).

## 4. Discussion

The prevalence of stunting, a chronic form of malnutrition, has been decreasing over the years, however, it remains above 23% nationally [18,19,20]. Although the prevalence of stunting in the present study was lower than the national prevalence at 8.5 and 11.6 in the control and intervention groups, respectively, it remains a cause for concern. Studies conducted in the Limpopo province focused on feeding practices and factors that contribute to infant malnutrition and not intervention strategies. For instance, a study by Mamabolo et al. [21], which investigated infant feeding practices and anthropometry at regular intervals (1, 3, 6, 9, and 12 months) in Mankweng, reported a stunting prevalence of 9.6%. This prevalence was attributed to maternal socioeconomic status, such as level of education, employment status, parity, and access to electricity.

On the other hand, Kleynhans et al. [22] reported a stunting prevalence of 18% in rural Limpopo province, which was influenced by low birth weight and early complementary feeding. Modjadji et al. [23], in a study conducted in the Waterberg district, reported that stunting was the prevalent form of malnutrition among children aged 12–23 months (62.4%), with delayed introduction of solid foods increasing the odds of stunting [23]. Similarly, Mphasha et al. reported that about 52.3% of mothers gave children solid food before six months [4]. The above studies highlighted the need for intervention strategies that will inform the feeding practices of infants. The present study revealed a positive impact of complementary feeding education programs on the nutritional status of infants. For instance, in the intervention group, the post-intervention prevalence of severe stunting, stunting, and underweight was reduced by 6.4%, 3.1%, and 23.7%, respectively, compared to the control group. The results of the present study highlight the critical role of targeted complementary feeding education programs in reducing infant malnutrition.

Undernutrition has been reported as an important indirect cause of child mortality, especially in low- and middle-income countries. However, the education of the mother about complimentary food was reported to have a positive impact in improving the nutritional status of infants [24]. However, factors such as affordability and access to food may limit the complementary food choices by the infants’ mothers. Hence, some studies recommended interventions that are culturally appropriate and that use locally available food [25,26]. The present study used a practical demonstration of preparing affordable, accessible, and culturally accepted foods using locally available ingredients, which proved effective. For instance, Sharma et al. [26] reported that the growth and supplemental feeding of children aged six months to one year in disadvantaged populations can be significantly enhanced by a community-based, culturally relevant nutrition education program that is provided through regular health care and digital tracking of undernourished children. Using locally available foods, the complementary food recommendations interventions improved mothers’ knowledge and children’s feeding practices and improved children’s nutrient intakes [25].

The role of educational intervention and its positive impact on caregivers knowledge relating to complementary feeding practices and child nutrition and growth have been reported globally [27,28]. In the present study, the intervention group showed notable gains in exclusive breastfeeding knowledge and improved understanding of complementary feeding, including knowledge of appropriate timing and reducing force-feeding practices and food hygiene. In addition, caregivers in the intervention group demonstrated better knowledge of food quantity, consistency, and frequency at end line. Furthermore, the control group’s knowledge score decreased by 4.3 units, while the intervention group improved by 13 units. A study by Lassi et al. [29] found similar findings to the present study. The study reported that caregivers who received educational interventions were more likely to follow recommended feeding practices, with a 62% higher uptake of recommended complementary foods compared to those in control groups [29]. A review and meta-analysis by Arigbo et al. [9] also reported those educational programs effectively improved timing, types, and amounts of complementary foods introduced to infants. Furthermore, this led to better hygiene practices [9]. Although the importance of educational interventions on caregivers’ knowledge cannot be overstated, there remains a gap that should be filled, especially in the Limpopo province. A cross-sectional study conducted in Seshego Township (a few kilometers from the present study’s site) reported that 33% of caregivers lacked appropriate infant and young child feeding knowledge [30]. Furthermore, the present study found no association between the mother’s anthropometric characteristics, such as weight and height, with weight and height for the age of the infant. This may suggest that the anthropometric development of the infant, such as gaining weight, is more dependent on the mother’s knowledge than the mother’s anthropometric characteristics. Therefore, more interventions should focus on providing complementary feeding practice knowledge for the mothers/caregivers.

This study used closed-ended questions, limiting participants’ responses, which could give vital information, and did not ask about caregivers’ opinions regarding cultural perceptions of complementary feeding. The data was too small to generalize about the complementary feeding knowledge in the Polokwane municipality because the focus was only on the tribal authorities, excluding mothers in the city and municipal villages outside tribal authorities. The present study did not measure food insecurity among the two groups, thus limiting the interpretation of the results as to whether the effect on growth was secondary to the intervention or availability/lack of food. Funding was also a factor in not covering a larger area. This study did not provide food to caregivers and depended on the food security of the household, meaning affordability, availability at home, and access to local markets. The follow-up was not rigorous; therefore, food consumption differed from one caregiver to another.

## 5. Conclusions

A community-based nutrition intervention program delivered through demonstration can effectively improve the nutritional status of children from six months to one year. The present study found that complementary feeding education programs had a positive impact on the prevention of stunting and underweight and were also found to have a positive impact on the normal growth patterns of the infant measured by MUAC, weight, and height to the age of the infant. We therefore recommend that community-based nutritional interventions should be incorporated into the public health profession systems and policies to mitigate the prevalence of undernutrition among infants. Furthermore, education is linked to the improvement of knowledge and skills; as such, the focus of the interventions should be on the development of new eating skills and changes in eating behavior and feeding styles.

## Figures and Tables

**Table 1:** Baseline characteristics of intervention and control groups.

Variable	Intervention: *n* = 95	Control: *n* = 94	*p*-Value
Sex			
Male	2 (2.1)	0 (0.0)	0.157
Female	93 (97.9)	94 (100)	
marital status			
Married	28 (29.5)	20 (21.3)	0.195
Unmarried	67 (70.5)	74 (78.7)	
Education level			
Primary and Secondary	78 (75.8)	77 (82.9)	0.587
Tertiary	23 (24.2)	17 (18.1)	
Occupation			
Unemployed	83 (87.4)	82 (87.2)	0.978
Employed	12 (12.8)	12 (12.8)	
Food security score			
Always enough to eat	53 (55.8)	29 (30.9)	0.002
Sometimes/often not enough to eat	42 (44.2)	65 (69.1)	
Type of house			
Informal dwelling	11 (11.6)	9 (9.6)	0.654
Formal dwelling	84 (88.4)	85 (90.4)	
Source of drinking water			
tap at home	45 (47.4)	48 (51.1)	0.054
communal tap/Borehole	21 (22.1)	28 (30.1)	
Buying	29 (30.5)	18 (19.1)	
Delivery method			
Caesarean section	34 (35.8)	19 (20.2)	0.017
Normal delivery	61 (64.2)	75 (79.8)	
Rubbish disposal	24 (25.3)	39 (41.5)	0.048
Municipality collects	71 (74.7)	55 (58.5)	
Pit at corner of the yard/other			
Baseline weight	Mean ± SD	Mean ± SD	
	3.0 ± 0.62 kg	3.1 ± 0.52 kg	
Birth length	Mean ± SD	Mean ± SD	
	48.1 ± 7.30 cm	48.9 ± 6.33 cm	

Data is presented in terms of frequency and percentages analyzed using the cross tabs option in SPSS.

**Table 2:** Difference in differences (DID) analysis for knowledge of caregivers and nutritional status.

Variables	Baseline		End Line		Difference		DID	*p*-Value
	Control	Intervention	Control	Intervention	Control	Intervention		
Knowledge	29.70 ± 0.83	25.10 ± 0.97	25.0 ± 0.99	38.10 ± 0.31	−4.73	13.0	17.72	<0.001
Weight	7.37 ± 0.12	6.69 ± 0.13	9.23 ± 0.48	10.40 ± 0.12	1.85	3.67	1.82	<0.001
Length	62.30 ± 0.79	61.00 ± 0.53	67.10 ± 0.98	73.60 ± 0.55	4.85	12.6	7.78	<0.001
Head circumference	41.9 ± 0.45	43.50 ± 2.07	42.80 ± 0.55	44.5 ± 0.17	0.905	1.07	0.16	0.950
MUAC	14.80 ± 0.32	14.10 ± 0.29	15.60 ± 0.24	16.70 ± 0.71	0.83	2.51	1.68	0.047

Data is presented in terms of mean ± standard deviation, mean difference, and DID. DID—difference-in-difference of the means for pre-test and end line in two groups using Two-Way Fixed Effects Model.

## Data Availability

This article is based on data collected from caregivers of infants 3 to 12 months of age from two tribal authorities within the Polokwane municipality, Limpopo province of South Africa. The data generated or analyzed during the current study is not openly accessible. However, it can be requested from the corresponding author.

## Supplementary Materials

The following supporting information can be downloaded at: https://www.mdpi.com/article/10.3390/children11121425/s1, Table S1: The CFEP comprised 11 main topics; Table S2: Anthropometric status of infants in control and experiment group at baseline and end line; Table S3: Training schedule/agenda; Table S4: Recipes prepared during intervention phase; Figure S1: The conceptual framework guiding the CEFP.

## References

[1] Mashaba R.G., Makwela M.S., Ntimana C.B., Seakamela K.P., Maimela E. (2024). Barriers and enablers to exclusive breastfeeding by mothers in Polokwane, South Africa. Front. Glob. Women’s Health.

[2] Lokossou G.A.G., Kouakanou L., Schumacher A., Zenclussen A.C. (2022). Human Breast Milk: From Food to Active Immune Response with Disease Protection in Infants and Mothers. Front. Immunol..

[3] Keikha M., Shayan-Moghadam R., Bahreynian M., Kelishadi R. (2021). Nutritional supplements and mother’s milk composition: A systematic review of interventional studies. Int. Breastfeed. J..

[4] Mphasha M.H., Makwela M.S., Muleka N., Maanaso B., Phoku M.M. (2023). Breastfeeding and Complementary Feeding Practices among Caregivers at Seshego Zone 4 Clinic in Limpopo Province, South Africa. Children.

[5] World Health Organization (2023). WHO Guideline for Complementary Feeding of Infants and Young Children 6–23 Months of Age.

[6] Stewart C.P., Iannotti L., Dewey K.G., Michaelsen K.F., Onyango A.W. (2013). Contextualising complementary feeding in a broader framework for stunting prevention. Matern. Child Nutr..

[7] Mutisya M., Kandala N., Ngware M.W., Kabiru C.W. (2015). Household food (in)security and nutritional status of urban poor children aged 6 to 23 months in Kenya. BMC Public Health.

[8] Policies and Guidelines—National Department of Health. https://www.health.gov.za/policies-and-guidelines/.

[9] Arikpo D., Edet E.S., Chibuzor M.T., Odey F., Caldwell D.M. (2018). Educational interventions for improving primary caregiver complementary feeding practices for children aged 24 months and under. Cochrane Database Syst. Rev..

[10] Mbhenyane X.G., Mandiwana T.C., Mbhatsani H.V., Mabapa N.S., Mushaphi L.F., Tambe B.A. (2023). Breastfeeding and complementary feeding practices of mothers exposed to the Baby-Friendly Hospital Initiative in Limpopo Province. South Afr. J. Child Health.

[11] Vir S.C. (2015). Public Health and Nutrition in Developing Countries (Part I and II).

[12] Lee R.D., Nieman D.C. (2010). Nutritional Assessment.

[13] Slovin E. (1960). Siovin’s Formula for Sampling Technique. https://prudencexd.weebly.com/.

[14] Spatial Without Compromise · QGIS Web Site. https://qgis.org/.

[15] Paintal K., Aguayo V.M. (2016). Feeding practices for infants and young children during and after common illness. Evidence from South Asia. Matern. Child Nutr..

[16] Fredriksson A., Oliveira G.M. (2019). de Impact evaluation using Difference-in-Differences. RAUSP Manag. J..

[17] Saville N.M., Shrestha B.P., Style S., Harris-Fry H., Beard B.J., Sen A., Jha S., Rai A., Paudel V., Sah R. (2018). Impact on birth weight and child growth of Participatory Learning and Action women’s groups with and without transfers of food or cash during pregnancy: Findings of the low birth weight South Asia cluster-randomised controlled trial (LBWSAT) in Nepal. PLoS ONE.

[18] Said-Mohamed R., Micklesfield L.K., Pettifor J.M., Norris S.A. (2015). Has the prevalence of stunting in South African children changed in 40 years? A systematic review. BMC Public Health.

[19] Wand H., Naidoo S., Govender V., Reddy T., Moodley J. (2024). Preventing Stunting in South African Children Under 5: Evaluating the Combined Impacts of Maternal Characteristics and Low Socioeconomic Conditions. J. Prev..

[20] Çelik M.N., Köksal E. (2024). Application of the infant and young child feeding index and the evaluation of its relationship with nutritional status in 6–24 months children. Rev. Nutr..

[21] Mamabolo R.L., Alberts M., Mbenyane G.X., Steyn N.P., Nthangeni N.G., Delemarre-van De Waal H.A., Levitt N.S. (2004). Feeding practices and growth of infants from birth to 12 months in the central region of the Limpopo Province of South Africa. Nutrition.

[22] Kleynhans I.C., MacIntyre U.E., Albertse E.C. (2006). Stunting among young black children and the socio-economic and health status of their mothers/caregivers in poor areas of rural Limpopo and urban Gauteng—The NutriGro Study. South Afr. J. Clin. Nutr..

[23] Modjadji P., Mashishi J. (2020). Persistent Malnutrition and Associated Factors among Children under Five Years Attending Primary Health Care Facilities in Limpopo Province, South Africa. Int. J. Environ. Res. Public Health.

[24] Imdad A., Yakoob M.Y., Bhutta Z.A. (2011). Impact of maternal education about complementary feeding and provision of complementary foods on child growth in developing countries. BMC Public Health.

[25] Fahmida U., Kolopaking R., Santika O., Sriani S., Umar J., Htet M.K., Ferguson E. (2015). Effectiveness in improving knowledge, practices, and intakes of “key problem nutrients” of a complementary feeding intervention developed by using linear programming: Experience in Lombok, Indonesia2. Am. J. Clin. Nutr..

[26] Sharma N., Gupta M., Aggarwal A.K., Gorle M. (2020). Effectiveness of a culturally appropriate nutrition educational intervention delivered through health services to improve growth and complementary feeding of infants: A quasi-experimental study from Chandigarh, India. PLoS ONE.

[27] Shi L., Zhang J. (2011). Recent Evidence of the Effectiveness of Educational Interventions for Improving Complementary Feeding Practices in Developing Countries. J. Trop. Pediatr..

[28] Dewey K.G., Adu-Afarwuah S. (2008). Systematic review of the efficacy and effectiveness of complementary feeding interventions in developing countries. Matern. Child Nutr..

[29] Lassi Z.S., Das J.K., Zahid G., Imdad A., Bhutta Z.A. (2013). Impact of education and provision of complementary feeding on growth and morbidity in children less than 2 years of age in developing countries: A systematic review. BMC Public Health.

[30] Muleka N., Maanaso B., Phoku M., Mphasha M.H., Makwela M. (2023). Infant and Young Child Feeding Knowledge among Caregivers of Children Aged between 0 and 24 Months in Seshego Township, Limpopo Province, South Africa. Healthcare.

