# Supplementary material for: The Effect of a Community-Based Complementary Feeding Education Program on the Nutritional Status of Infants in Polokwane Municipality, Limpopo Province, South Africa
Source: Children (Basel). 2024 Nov 26;11(12):1425. doi: 10.3390/children11121425 (PMC11675079; doi:10.3390/children11121425)
Supplement: Supplementary file 1 [file children-11-01425-s001.zip › children-3283286-supplementary.pdf]

Supplementary File

The Effect of a Community-Based Complementary Feeding Education Program on the Nutritional Status of Infants in Polokwane Municipality, Limpopo Province, South Africa

Maishahataba Solomon Makwela [1,*], Lindelani Fumulani Mushaphi [2] and Lufuno Makhado [2]

[1] Department of Human Nutrition and Dietetics, Faculty of Health Sciences, University of Limpopo, Polokwane 0727, South Africa
[2] Department of Nutrition, School of Health Sciences, University of Venda, Thohoyandou 0950, South Africa; lindelani.mushaphi@univen.ac.za (L.F.M.); lufuno.makhado@univen.ac.za (L.M.)
* Correspondence: maishataba.makwela@ul.ac.za

Table S1. The CFEP comprised 11 main topics.

Lesson 1:	The importance of exclusive breastfeeding during the first six months.
Lesson 2:	How to feed a sick baby less than six months of age.
Lesson 3:	Good hygiene practices prevent disease.
Lesson 4:	Starting complementary feeding at six months.
Lesson 5:	Complementary feeding from 6 up to 9 months.
Lesson 6:	Complementary feeding from 9 up to 12 months.
Lesson 7:	Complementary feeding from 12 to 24 months.
Lesson 8:	Food variety/variety of food.
Lesson 9:	How to add Micronutrient Powders to complementary Foods.
Lesson 10:	Feeding a sick child more than six months of age and feeding a non-breastfed child from 6 up to 24 months.

Table S2. Anthropometric status of infants in control and experiment group at baseline and end line.

Variables	Baseline: *n* (%)			End Line: *n* (%)		
	Control *n* = 94	Intervention *n* = 95	*p*-Value	Control *n* = 71	Intervention *n* = 77	*p*-Value
**Length for age**						
Severe stunting	8(8.5)	11(11.6)		1(1.4)	4(5.2)	
Stunted	22(23.4)	40(42.1)	0.108	26(36.7)	30(39.0)	0.082
Normal height	64(68.1)	44(46.4)		37(52.1)	28(36.4)	
**Weight for length**						
Severe wasting	3(3.2)	2(2.1)		0(0.0)	0(0.0)	
Wasted	5(6.4)	4(5.3)		2(2.8)	3(3.9)	
Normal WHZ	34(36.2)	47(49.5)	0.451	22(31.0)	24(31.2)	0.864
At risk of overweight	25(26.6)	25(26.3)		31(43.7)	32(41.6)	
Overweight & obese	26(27.6)	16(16.8)		16(22.5)	18(23.4)	
**Weight for age**						
Severe underweight	0(0.0)	1(1.1)		0(0.0)	1(1.3)	
Underweight	10(10.6)	25(26.3)	0.017	4(5.6)	2(2.6)	0.636
Normal WAZ	48(51.1)	52(54.7)		38(53.5)	44(57.1)	
Possible growth problem	36(38.3)	19(17.9)		29(40.8)	27(39)	

Table S3. Training schedule/agenda.

Time	Activity	Presenter
**Day 1**		
**Session 1**		
08h30–09h00	Registrations	Mothers
09h30–10h00	Introductions and expectations	Makwela
10h00–10h20	Identification of education cards	Makwela
**Session 2**		
10h20–11h00	Components of complementary feeding	Makwela
11h00–11h15	Ice breaker—song on complementary feeding	Makwela
11h15–11h30	Snack break	
**Session 3**		
11h35–13h00	The importance of continued breastfeeding after 6 months	Makwela
	What we should consider when thinking of complementary feeding for each age group	Makwela
	Starting Complementary Feeding when the infant reaches 6 months	Makwela
13h00–1400	Lunch	
14h00–15h00	Cooking session	
**Day 2**		
08h30–09h00	Complementary feeding from 6 up to 9 months	Makwela
09h30–10h00	Complementary feeding from 9 up to 12 months	Makwela
10h00–10h20	Complementary feeding from 12 up to 24 months	Makwela
10h20–10h45	Snack break	
10h45–11h20	Non-breastfed child from 6 up to 24 months	Makwela
11h20–12h50	Food Variety of food	Makwela
	How to Add fortified foods to Complementary feeding	
11h50–12h20	Feeding the sick child more than 6 months of age	Makwela
**Session 4**		
Self-study	Regular growth monitoring and promotion	Makwela
	When to take your child to the health facility	Makwela

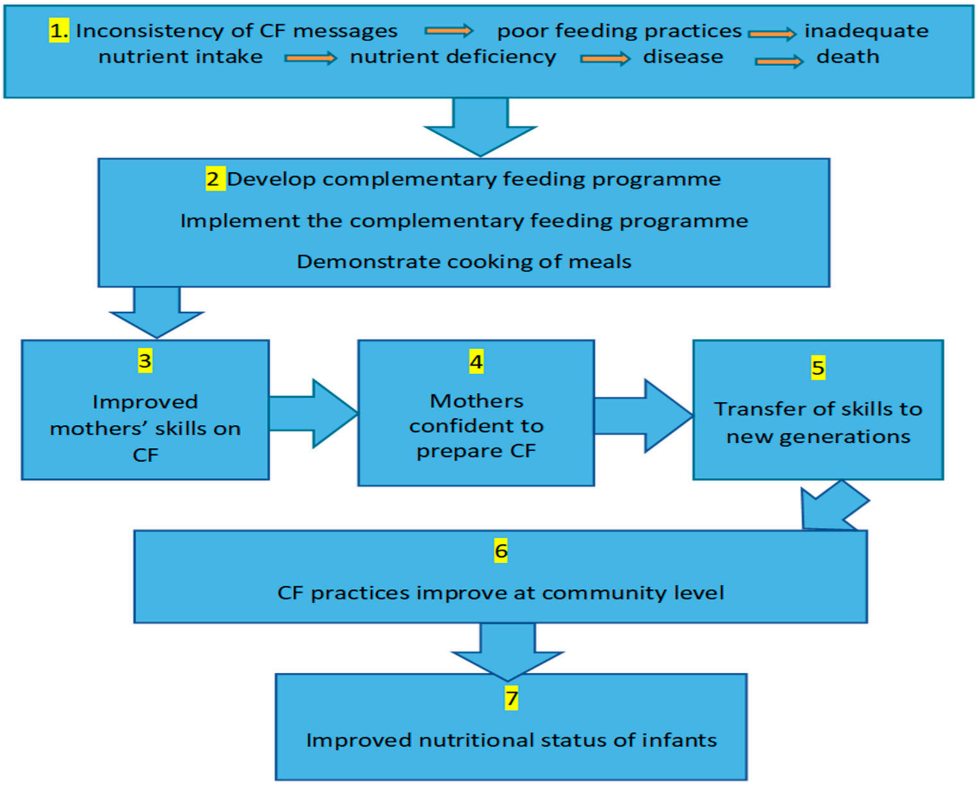

Figure S1. The conceptual framework guiding the CEFP.

Stage 1: The consistency of CF messages

Evidence suggests that the messages provided at health care facilities during antenatal care period and postnatal are given at random and are not the same during each presentation. Some of the caregivers may miss the only messages that are communicated since they don't all attend at similar days. Furthermore, some women are first time mothers who are without anyone to assist them on how to prepare and feed complementary food timeously, adequately, variety not catered for and the frequency of feeding. The inconsistent messages may lead caregivers to use instinct when feeding their infants and may lead to inadequate or overly intake of nutrients. Both the latter may result in malnutrition, either in the form of undernutrition or overnutrition. In majority of cases, undernutrition presents because of poor infant feeding practices. The current undernutrition reported in south Africa is stunting (27%) which has been prevalent for the past two decades (SADHS, 2016). Under five overweight has increased decreased from 10.4% to 11.3 between 2004 and 2017 respectively (UNICEF, WHO, World Bank,2021) which is above the global target of 10%.

Stage 2: Development and implementation of CF program

Stage 2 was discussing the development and implementation of a complementary feeding program respectively (2.1 and 2.2)

Stage 2.1: Development of CF program

The study is aimed at developing a complementary feeding program which is intended to educate pregnant and lactating mothers with consistent evidence-based messages that would reduce inconsistency of CF messages. It focused on the importance of timeous, varied, adequate, frequent, and safe complementary feeding. The CF education program developed to address the information gap by educating mothers and caregivers about the need to introduce complementary feeding and

how to prepare the CF food so that they don't only learn theory but also practical of preparations through demonstration. Caregivers were allowed to prepare Cf using developed recipes under supervision of the researcher, and challenges were addressed. The recipes were based on locally available food which are culturally acceptable to communities. The development of the CF program was based on Guiding principles for complementary feeding of the breastfed child by Dewey (2001) and Indicators for assessing infant and young child feeding practices (Part 2), by WHO (2010).

Stage 2.2: Implementation of the Complementary feeding education program

At this stage the complementary feeding education programme was implemented on caregivers in the experimental group. Those caregivers who consented to participate were enrolled and kept updated with the dates of the training program. For this stage the health believe model model was adopted to educate the caregivers. The transtheoretical model is suitable for the study based on its stages of preparing the caregivers (to be explained in full). In summary it considers the fact that some caregivers still need to be prepared to act, then taught on how to start, support them on how to continues with the behavior and it becomes a cyclical process. Caregivers were taught how to prepare recipes, which gave an opportunity to identify and address their barriers.

Stage 3,4,5: Improved maternal skills, confidence, and skills transfer

Implementation of the developed CF program may lead to improvement of skills to prepare CF and improve the nutritional status of infants. This process has a potential to improve on the skills of and caregiver's confidence to prepare. There is a good potential for caregivers who find this interesting to transfer the learned skills to their young ones, family and subsequently the rest of the community. If caregivers are convinced that improved nutritional status of infants are a result of complementary feeding education and practices, are more likely to appraise feeding practices and share the skill with younger generations of family members, friends, and some members of the community.

Stage 6,7: Improved CF skills at community level

The implementation of the leaned skills by caregivers, caregivers and communities is more likely to improve the CF practices. When the community is skilled and practice appropriate CF, feeding practices were done in line with infant feeding guidelines, therefore infants are likely to meet the MAD for that community, if majority of infants meet the MAD, subsequently nutritional status of infants may improve. The stunting (27%) and obesity rates of (4.5%) in the country may also reduce to acceptable levels and eventually be eradicated. The current MAD in South Africa is at 16% (SADHS, 2016), which is far below the national target of 50%. The following section will discuss the consequences of lack of intervention to address malnutrition.

Table S4. Recipes prepared during intervention phase.

Annexure: Recipe booklet

Recipe booklet

Table of Contents

Recipe 1 : Maizemeal porridge 5
Recipe 2 : Blended Chicken Stew, Rice And Sweet Potatoes....................5
Recipe 3: Mabele (Sorghum) Porridge....................7
Recipe 4: Scrambled Eggs 7
Recipe 5: Samp And Beans ,Cooked Carrots Blend 7
Recipe 6 : Samp And Beans 8
Recipe 7: Mince, Macaroni And Green Beans mix....................8
Recipe 9: Pilchard Fish, Mashed Potatoes And Beetroot Salad Blended 11
Recipe 10 : Stew Beef, Rice And Cabbage mix 12
Recipe 11: Chicken Livers, Pap And Cooked Morogo/Spinach 13
Recipe 12: Bean , Lentil Stew And Mealie Rice 14
Recipe 13: Macaroni and Cheese 15

Recipe 1 : Maizemeal porridge

MAIZE MEAL PORRIDGE	
**Serving size**<br>• 6 – 8 months: 3 tbsp- ¼ cup<br>• 9 – 12 months: ½ -1 cup	
**Ingredients**	**Portion size**
Maize meal	25g
Water	100ml
**Method**	
1. Mix maize meal with a little cold water to make a paste.<br>2. Bring the rest of water to the boil in a pot.<br>3. Stir maize meal paste into boiling water.<br>4. Simmer until cooked, 45 – 60 minutes.<br>5. Serve.	

***tsp- teaspoon**

Recipe 2 : Blended Chicken Stew, Rice And Sweet Potatoes

CHICKEN STEW	
**Serving size**<br>• 6 – 8 months: 3 tbsp- ¼ cup (blended chicken stew, rice and sweet potatoes)<br>• 9 – 12 months: ¼ cup	
**Ingredients**	**10 portions**
Chicken	500g
Oil	15ml (1 tbsp)

Onions	1 medium
Carrots	1 medium
Green beans	200g
Soup, cream of chicken	½ pkt
**Method**	

1. Clean chicken, remove excess fat and cut into small pieces.
2. Place chicken pieces in a pot with just enough water to bring to boil and cook until chicken is cooked.
3. Drain the remaining water from the chicken and reserve it. Let chicken pieces cool.
4. Prepare vegetables; peel and chop onion, peel and chop carrots, top and tail and chop green beans.
5. Add oil to pot and heat, add onion and brown.
6. Add other vegetables, prepared chicken and reserved cooked chicken water and simmer until vegetables are tender.
7. Mix soup with cold water, add to the chicken and simmer for 5 minutes.

Serve.

RICE

Serving size

- 6 – 8 months: 3 tbsp- ¼ cup (blended chicken stew, rice and sweet potatoes)
- 9 – 12 months: ¼ cup

**Ingredients**	**10 portions**
Rice	1kg
Salt (optional)	¼ tsp
Water	700ml

Metho

1. 1n a pot with a good fitting lid bring water to a boil.
2. Add rice and salt and stir.
3. Cover and reduce heat to medium low. You will know that your temperature is correct if a little steam is visible leaking from the lid. A lot of steam means your heat is too high.
4. Cook for 30 minutes.
5. DO NOT LIFT LID.
6. Remove from heat and fluff with fork.

Serve.

SWEET POTATOES

Serving size

6 – 8 months: 3 tbsp- ¼ cup (blended chicken stew, rice and sweet potatoes)

9 – 12 months: ¼ cup

**Ingredients**	**10 portions**
Sweet potatoes	600g (4 medium)
Boiling water	1 cup

Method

1. Peel, wash and cut into cubes as near to cooking time as possible.
2. Place peeled, cubed sweet potatoes in boiling water, until water dries.
3. Mash together.
4. Serve.

Recipe 3: Mabele (Sorghum) Porridge

MABELE (SORGHUM) PORRIDGE	
**Serving size**<br>• 6 – 8 months: 3 tbsp- ¼ cup<br>• 9 – 12 months: ½ -1 cup	
**Ingredients**	**1 portions**
Mabele (sorghum)	25g
Water	150ml
**Method**	

1. Mix mabele (soghurm) with a little cold water to make a paste.
2. Bring rest of water to the boil in a pot.
3. Stir mabele (soghurm) paste into boiling water.
4. Simmer until cooked, 25 – 30 minutes.
5. Serve.

Recipe 4: Scrambled Eggs

SCRAMBLED EGGS	
**Serving size**<br>• 6 – 8 months: 2 tsp<br>• 9 – 12 months: 1 tbsp	
**Ingredients**	**10 portions**
Eggs	1 large
Milk	15 ml
Salt	¼ tsp
Cooking oil	15 ml (1 tbsp)
**Method**	

1. Crack eggs into a medium bowl. Add milk and salt; whisk until combined.
2. Heat oil in a large pan over medium heat just until it's hot, swirling the pan to coat the bottom and sides.
3. Add eggs mixture and cook, stirring slowly but continuously, until they just begin to thicken, about ½ a minute (30 seconds)
4. Begin vigorously stirring the eggs with a flat-edged wooden spatula, scraping the bottom of the pan as you go.
5. When eggs are just barely cooked through, after another 1 to 2 minutes or so, remove the pan from the heat.
6. Serve.

Recipe 5: Samp And Beans ,Cooked Carrots Blend

COOKED CARROTS	
**Serving size**	
**Ingredients**	**10 portions**
Fresh carrots	60g (1 medium)

Boiling water	125 ml ¼ cup
**Method**	
1. Wash, scrape and cut into slices as near to cooking time as possible<br>2. Place peeled, sliced carrots in boiling water, until water dries or until tender<br>3. Add into cooked samp and beans mix	

Recipe 6 : Samp And Beans

**Serving size**<br><br>6 – 8 months: 3 tbsp- ¼ cup (blended samp and beans and cooked carrots)<br><br>9 – 12 months: ¼ cup	
**Ingredients**	**1 portions**
Samp	½ cup
Beans, dry	¼ cup
Cooking oil	15ml (1 tbsp)
Onion	1 small
Tomatoes	1 small
Salt	¼ tsp
**Method**	
1. Cover samp with water, set aside to soak overnight.<br>2. Cover beans with water, set aside to soak overnight.<br>3. In the morning drain beans and place in a pot with water, bring to the boil. Allow to boil for 20 minutes.<br>4. Drain samp. Drain beans.<br>5. Combine samp and beans and water and boil until tender (do not add salt).<br>6. Heat oil in a pan.<br>7. Chop onions and tomatoes.<br>8. Add onions to the oil and cook until brown, add tomatoes and cook for 5 minutes.<br>9. Stir salt into onion mixture and add this to the samp and beans.<br>9. Simmer for 5 to 10 minutes.<br>10. Serve.	

Recipe 7: Mince, Macaroni And Green Beans mix

**SAVOURY MINCE**	
**Serving size**<br><br>• 6 – 8 months: 3 tbsp- ¼ cup (mince, macaroni and green beans blended)<br>• 9 – 12 months: ¼ cup	
**Ingredients**	**10 portions**
Onions	1 small
Cooking oil	5ml (1 tbp)
Mince	50g
Curry powder	¼ tsp
Salt	¼ tsp
Water	50 ml
**Method**	

GREEN BEANS	
**Serving size**<br>6 – 8 months: 3 tbsp- ¼ cup (mince, macaroni and green beans blended)<br>9 – 12 months: ¼ cup	
**Ingredients**	**1 portions**
Onions	½ medium
Green beans	50g
Salt	½ tsp
Margarine, yellow, brick	5g
**Method**	
Peel and dice onions.<br>Wash top, tail and slice green beans.<br>Bring a pot with a little water to the boil.<br>Add onion, green beans and salt to the water and bring to the boil.<br>Cook until water dries or until just tender.<br>Add margarine and stir to mix.<br>Serve.	

1. Peel and finely chop onions.
2. Heat cooking oil in pot, add onions and fry.
3. Add mince to onions and fry.
4. Once browned, add curry and salt to taste, add water and simmer for 20 minutes.
5. Serve.

MACARONI	
**Serving size**<br>• 6 – 8 months: 3 tbsp- ¼ cup (mince, macaroni and green beans blended)<br>• 9 – 12 months: ¼ cup	
**Ingredients**	**1 portions**
Water	250ml (cup)
Salt (optional)	¼ tsp
Macaroni	250g (1 cups)
Cooking oil	½ tsp
**Method**	
1. n a pot with a good fitting lid bring water to a rolling boil.<br>2. Add salt (optional)<br>3. Add macaroni and stir.<br>4. Boil macaroni for 10 minutes or until tender but firm.<br>5. Remove from heat and drain excess water.<br>6. Sprinkle with oil and fluff with fork.<br>7. Serve.	

Recipe 8: Lentil Stew, Rice And Pumpkin/Butternut Blend

LENTIL STEW	
**Serving size**<br>6 – 8 months: 3 tbsp- ¼ cup (lentil stew, rice and pumpkin/butternut blend)<br>9 – 12 months: ¼ cup	
**Ingredients**	**1 portions**
Lentils	100g

Onion	½ small
Carrots	1 medium
Barbeque spice(exclude at six months)	½ tsp
Tomatoes	1/2 small
Soup, tomato	½ pkt
**Method**	
Cover lentils with hot water, and leave to soak while preparing other ingredients.<br>Peel and chop onion, peel and grate carrot. Combine with water and cook over medium heat until onion is soft.<br>Wash and chop the tomatoes.<br>Stir in the barbeque spice to onion and carrot mix. Cook for 1 – 2 minutes, stirring all the time.<br>Add chopped tomatoes.<br>Drain lentils and add to the cooked mix and add enough water to cover.<br>Simmer for about 20 – 30 minutes, or until lentils are cooked.<br>Mix soup powder with a little cold water; pour into the lentil mix, stirring all the time.<br>Simmer for 3 to 5 minutes.<br>Add extra hot water if needed.<br>Serve	

PUMPKIN/BUTTERNUT	
**Serving size**<br>6 – 8 months: 3 tbsp- ¼ cup (lentil stew, rice and pumpkin/butternut blend)<br>9 – 12 months: ¼ cup	
**Ingredients**	**1 portion**
Pumpkin/butternut	60g (1 small)
Margarine, yellow, brick	5g
**Method**	
Wash pumpkin/butternut, peel, remove seeds, and slice into blocks (20x20x20mm).<br>Bring small amount of water to boil in pot.<br>Add pumpkin/butternut to pot and bring to boil, the water must only just cover the pumpkin/butternut.<br>Cook until water dries or just tender.<br>Add margarine, mix and stir.<br>Serve.	

Recipe 9: Pilchard Fish, Mashed Potatoes And Beetroot Salad Blended

PILCHARD FISH	
**Serving size**<br>6 – 8 months: 3 tbsp- ¼ cup (pilchard fish, mashed potatoes and beetroot salad blended)<br>9 – 12 months: ¼ cup	
**Ingredients**	**10 portions**
Onion	½ medium
Cooking oil	30ml (2 tbsp)
Tomatoes	2 medium
Pilchard fish	1 tin (400g)
Salt	½ tsp
**Method**	
Peel and finely chop onions.<br>Heat cooking oil in pot, add onions and fry.<br>Once onions are browned, add tomatoes and cook until tomatoes soften.<br>Add pilchard and salt to taste and simmer for 5 to 10 minutes.<br>Serve.	

MASHED POTATOES	
**Serving size**<br>6 – 8 months: 3 tbsp- ¼ cup (pilchard fish, mashed potatoes and beetroot salad blend)<br>9 – 12 months: ¼ cup	
**Ingredients**	**10 portions**
Potatoes	60g (1 medium)
Salt	½ tsp
Margarine, yellow, brick	5g

Milk	50ml
**Method**	
Bring water in pot to boil.<br>Wash and peel potatoes, cut into quarters.<br>Add potatoes to boiling water, and cook until tender (test with a fork).<br>Drain water from potatoes, keeping some aside for if needed in mashing.<br>Add salt and margarine to potatoes and stir to mix.<br>Add milk and mash<br>Add extra water if needed and mash till consistency is even.<br>Serve.	

**BEETROOT SALAD**	
**Serving size**<br>6 – 8 months: 3 tbsp- ¼ cup (pilchard fish, mashed potatoes and beetroot salad blend)<br>9 – 12 months: ¼ cup	
**Ingredients**	**10 portions**
Beetroot	600g (4 medium)
Vinegar	2 tbsp
Sugar	2 tbsp
**Method**	
Prepare beetroot by washing it and slicing off leaves and long root, but leaving a 1cm stub of each.<br><br>Place in pot with water to cover beetroot and boil.<br>Simmer on medium heat until tender (test with a fork).<br>Drain water and leave beetroot to cool.<br>Peel and grate or slice.<br>Mix other ingredients with beetroot.<br>Chill until needed.<br>Serve.	

Recipe 10 : Stew Beef, Rice And Cabbage mix

**BEEF STEW**	
**Serving size**<br>6 – 8 months: 3 tbsp- ¼ cup (Beef Stew, Rice And Cooked Cabbage blend))<br>9 – 12 months: ¼ cup	
**Ingredients**	**10 portions**
Onions	1 medium
Potatoes	1 medium
Tomatoes	2 medium
Cooking oil	15ml (1 tbsp)
Beef	500g
Salt	½ tsp
Water	$^{1}/_{3}$ cup (80ml)
**Method**	

COOKED CABBAGE	
**Serving size**<br>6 – 8 months: 3 tbsp- ¼ cup (Beef Stew, Rice And Cooked Cabbage blend)<br>9 – 12 months: ¼ cup	
**Ingredients**	**10 portions**
Cabbage	½ medium
Water	¼ cup (60ml)
Cooking oil	15ml (1 tbsp)
Salt	¼ tsp
**Method**	
Rinse cabbage.<br>Shred the cabbage and put in a pot.<br>Add water and simmer until soft.<br>Add cooking oil and salt.<br>Braise the cabbage, it should not be browned.<br>Serve.	

Wash, peel and chop vegetables coarsely.<br>Heat the oil in a pot.<br>Cut beef into small cubes, add to heated oil and fry until meat is browned.<br>Add onions, potatoes and tomatoes, fry and stir occasionally.<br>Add salt and water to the meat mixture and bring to boil.<br>Simmer until tender: 60 to 90 minutes<br>Serve

COOKED CABBAGE

Recipe 11: Chicken Livers, Pap And Cooked Morogo/Spinach

CHICKEN LIVERS	
**Serving size**<br>6 – 8 months: 3 tbsp- ¼ cup (Chicken Livers, Pap And Cooked Morogo/Spinach)<br>9 – 12 months: ¼ cup	
**Ingredients**	**10 portions**
Onions	14 small
Tomatoes	1/4 small
Cooking oil	5ml (1 tsp)
Chicken livers	50g
Chicken spice	¼ tsp
Salt	¼ tsp
**Method**	

Wash, peel and chop onions and tomatoes.<br>Wash chicken livers and cut them to half.<br>Heat the oil in a steam pot.<br>Add chicken spice and salt to chicken livers.<br>Add chicken livers to heated oil and fry until brown.<br>Add onions and tomatoes and stir occasionally.<br>Simmer until livers are soft and tender, about 15 minutes.<br>Serve.

**PAP**	
**Serving size**<br>6 – 8 months: 3 tbsp- ¼ cup (Chicken Livers, Pap And Cooked Morogo/Spinach)<br>9 – 12 months: ½ -1 cup	
**Ingredients**	**10 Portions**
Maize meal	3 cups
Salt (optional)	¼ tsp
Water	1.5L
**Method**	
Bring water to boil in a pot.<br>Add salt (optional) and maize meal while stirring to avoid lumps to form.<br>Stir thoroughly until smooth and cover with a lid.<br>Reduce heat and simmer for 35 minutes while stirring occasionally.<br>Serve.	

**COOKED MOROGO/SPINACH**	
**Serving size**<br>6 – 8 months: 3 tbsp- ¼ cup (Chicken Livers, Pap And Cooked Morogo/Spinach)<br>9 – 12 months: ¼ cup	
**Ingredients**	**10 portions**
Onion	½ medium
Morogo/spinach	500g
Salt	½ tsp
Margarine, yellow, brick	25g
**Method**	
Wash morogo/spinach.<br>Bring a pot with very little water to the boil.<br>Add onion, morogo/spinach and salt to the water.<br>Stir with a wooden spoon until all morogo/spinach has wilted.<br>Cover and allow to cook for 3 -5 minutes.<br>Add margarine and stir to mix.<br>Serve.	

Recipe 12: Beans, Lentils Stew And Mealie Rice

**BEAN AND LENTIL STEW**	
**Serving size**<br>6 – 8 months: 3 tsp- ¼ cup (bean , lentil stew and mealie rice)<br>9 – 12 months: ¼ cup	
**Ingredients**	**10 portions**
Dried sugar beans	250g (1 cup)

Oil	15ml (1 tbsp)
Onion	1 medium
Carrots	1 medium
Water	2L
Dried mixed herbs	½ tsp
Brown lentils	250g (1 cup)
Tomatoes	2 medium
Salt	½ tsp
**Method**	
Soak beans in water overnight. Rinse and drain.<br>Wash, peel and finely chop onions<br>Wash, scrape, and grate carrots<br>Wash and chop tomatoes<br>Heat oil in a large pot and fry carrots and onions.<br>Add the water, dried herbs, lentils and beans.<br>Bring to the boil and reduce the heat. Simmer with a lid for 2-2½ hours or until the beans are tender.<br>Add tomatoes and salt and simmer for another 15 minutes or until tomatoes are soft.<br>Serve.	

**MEALIE RICE**	
**Serving size**<br>6 – 8 months: 3 tsp- ¼ cup (bean, lentil stew and mealie rice)<br>9 – 12 months: ½ -1 cup	
**Ingredients**	**10 portions**
Mealie rice	1 cup
Salt (optional)	¼ tsp
Water	1L
**Method**	
Rinse the mealie rice and drain off water.<br>Add water to cover mealie rice in a pot and bring to boil.<br>Reduce heat and simmer for 45 - 60 minutes while stirring occasionally.<br>Add salt (optional) and stir.<br>Serve.	

Recipe 13: Macaroni and Cheese

**MACARONI AND CHEESE**	
**Serving size**<br>6 – 8 months: 3 tsp- ¼ cup<br>9 – 12 months: ¼ cup	
**Ingredients**	**10 portions**
Water	1L
Salt	¼ tsp
Macaroni	500g (2 cups)
Margarine	1 tbsp
Flour	1 tbsp
Salt	¼ tsp
Milk	250 ml (1 cup)

Shredded cheddar cheese	½ cup
**Method**	
In a pot with a good fitting lid bring water to a rolling boil.<br>Add salt (optional)<br>Add macaroni and stir.<br>Boil macaroni for 10 minutes or until tender but firm.<br>Remove from heat and drain excess water. Set aside<br>In a saucepan, heat margarine in medium heat until melted.<br>Stir in flour, salt and slowly add milk.<br>Stir all the time until bubbly.<br>Stir in cheese until melted.<br>Add to cheese sauce to macaroni; stir to combine.<br>Serve immediately.	
